# Longitudinal Monitoring of the Development of Antifilarial Antibodies and Acquisition of *Wuchereria bancrofti* in a Highly Endemic Area of Haiti

**DOI:** 10.1371/journal.pntd.0001941

**Published:** 2012-12-06

**Authors:** Katy L. Hamlin, Delynn M. Moss, Jeffrey W. Priest, Jacquelin Roberts, Joseph Kubofcik, Katherine Gass, Thomas G. Streit, Thomas B. Nutman, Mark L. Eberhard, Patrick J. Lammie

**Affiliations:** 1 Centers for Disease Control and Prevention, Atlanta, Georgia, United States of America; 2 National Institutes of Health, Bethesda, Maryland, United States of America; 3 The LF Support Center at the Task Force for Global Health, Decatur, Georgia, United States of America; 4 The Notre Dame Haiti Program, University of Notre Dame, Notre Dame, Indiana, United States of America; Michigan State University, United States of America

## Abstract

Antifilarial antibody testing has been established as a sensitive and specific method of diagnosing lymphatic filariasis. However, the development of serological responses to specific filarial antigens and their relationship to acquisition of infection is poorly understood. In order to evaluate whether the development of antigen specific antifilarial antibodies precedes microfilaremia and antigenemia, we compared the antibody responses of serum samples collected between 1990 and 1999 from a cohort of 142 Haitian children followed longitudinally. Antigen status was determined using the Og4C3 ELISA and the presence of microfilaremia was detected using microscopy. Antibody responses to Wb123, a *Wuchereria bancrofti* L3 antigen, were measured using a Luciferase Immunoprecipitation System (LIPS) assay. Antibody responses to Bm14 and Bm33, *Brugia malayi* antigens and to a major surface protein (WSP) from Wolbachia were analyzed using a multiplex bead assay. Over follow-up, 80 (56%) of the children became antigen-positive and 30 (21%) developed microfilaremia. Detectable antibody responses to Bm14, Bm33, Wb123, and WSP developed in 95%, 100%, 92%, and 29% of children, respectively. With the exception of WSP, the development of antibody responses generally preceded detection of filarial antigen. Our results show that antifilarial antibody responses can serve as an important epidemiological indicator in a sentinel population of young children and thus, may be valuable as tool for surveillance in the context of lymphatic filariasis elimination programs.

## Author Summary

Programs to eliminate lymphatic filariasis (LF) are designed to interrupt transmission of the parasite by treating the human reservoir of infection. As infection levels decline, assessing infection and transmission levels becomes more and more challenging. In principle, measuring the level of antibody to filarial antigens in children may provide a sensitive measure of transmission intensity. Here, we used samples collected over time from 142 Haitian children living in an area of intense transmission of LF to determine when they first developed antibody responses to defined filarial antigens compared to when they became infected. Antibody responses were measured to several filarial antigens using sensitive assays based on multiplex and LIPS assay methods. Our results show that antibody responses developed before infection could be detected by conventional tests for the presence of microfilariae or antigen in the blood. These results support the idea that antibody tests can be used to monitor the impact of mass drug administration programs on transmission of LF and to carry out surveillance for LF after drug treatments have stopped.

## Introduction

Lymphatic filariasis (LF) is a significant cause of global morbidity and is responsible for causing lymphedema, elephantiasis, and hydrocele. Research focusing on the pathogenesis of LF has historically neglected children, both because the onset of clinical disease tends to occur in adults and due to the logistical and ethical issues involved with including children in studies; however, surveys in areas of intense transmission demonstrate that children acquire infections early in life [1], [2]. In addition, recent studies have demonstrated that lymphangiectasia generally starts in early childhood and have documented the presence of significant subclinical pathology in children [3], [4], dispelling the belief that disease manifestations are restricted to adulthood. More important from the public health perspective, there is now evidence that early disease in children is reversible following treatment [5]. These observations reinforce the argument for using community-based treatment strategies for the control and elimination of LF as such efforts will prevent the development of morbidity in children residing in LF-endemic areas as well as in future generations [6].

The World Health Organization estimates that there are 120 million people living in 72 countries that are infected with the filarial parasite which causes LF and 1.34 billion people worldwide who live in filariasis-endemic areas and are at risk of developing the infection [7], [8]. Mass drug administration (MDA) programs have now been developed in more than 50 countries and more than ten countries have stopped MDA in all or part of the country after carrying out 5 or more rounds of annual MDA [8]. These successes in the efforts to eliminate LF have highlighted the need for more sensitive, standardized tools to help programs define MDA endpoints and to conduct surveillance [9], [10]. Currently, WHO guidelines are based on the monitoring of antigenemia in children; however, since antibody responses generally develop before patent infection, their detection in a serum-based assay could be used to provide an early measure of filarial exposure and ongoing transmission [10], [11].

Monitoring the natural history of LF is important in defining the relationship between the development of antibody responses to specific filarial antigens and the acquisition of infection. Longitudinal studies of the development of antifilarial antibody responses in a population of children provide an opportunity to compare the performance of different diagnostic tools relative to the first detection of microfilaremia and antigenemia. Such studies can help inform our choices of tools best suited for monitoring transmission and conducting post-MDA surveillance. In this study, we monitored the development of antifilarial immunity in a cohort of Haitian children living in a highly endemic area before the onset of MDA campaigns.

## Materials and Methods

### Study location

The study population and design have been previously described [1]. In brief, children were followed longitudinally to investigate risk factors for filarial infection. The children were residents of Leogane, Haiti, a coastal community with a population of approximately 10,000–15,000 people that is known to be highly endemic for lymphatic filariasis [12], [13]. Although a small number of persons were treated as part of drug studies [14], no MDA programs were conducted in the community over the period of follow-up (1990–1999) nor was diethylcarbamazine available except through Ste. Croix Hospital. Neighborhoods that were known to have a prevalence of microfilaremia greater than 20% were targeted for the longitudinal study.

### Study population

The protocol for this study was reviewed and approved by the Centers for Disease Control and Prevention Institutional Review Board and the Ethical Committee of Ste. Croix Hospital (Leogane, Haiti). Mothers of children less than 24 months of age were approached for participation in the study on a rolling basis. After explaining the purpose of the study in Creole, the mothers were asked to provide consent for themselves and for their children to be enrolled in the study. Consent was documented verbally at enrollment and during follow up for all study participants; children seven years of age or older provided assent. Consent and assent were documented by project staff on enrollment forms. Verbal consent was approved by the IRB and Ethical Committee because of the low rate of literacy in the communities that were being monitored. Children who were enrolled were assessed annually over the period of follow-up (1990–1999) for microfilaremia, antigenemia, and intestinal parasite burden. Serum samples from each study visit were stored for lab analysis. The frequency of follow-up was influenced by political events which limited field work. Those children with 5 or more serum samples at the conclusion of the study were included in the current cohort and their serum samples were selected for antigen and antifilarial antibody testing. Samples with incomplete information and duplicate samples were excluded from the analyses.

### Parasitologic exams

Parasitologic examinations were performed as previously described [1]. Briefly, a nocturnal blood exam (Giemsa-stained 20 ul-thick film) was performed on the children and their mothers in order to determine the baseline microfilaremia infection status [15]. Follow-up examinations for microfilaremia occurred every 9–12 months, and stool examinations were performed at regular intervals to monitor intestinal parasite burdens in the children. Stools were preserved in 10% formalin and were examined for ova and parasite following concentration by the formalin/ethyl acetate technique. When infections were detected, microfilaremic persons were treated with a single dose of diethylcarbamazine (DEC; 6 mg/kg) and children with *Ascaris, Trichuris*, or hookworm infections were provided treatment with mebendazole (100 mg×3 days).

### Serological assays

Serum samples (100 ul) collected during follow-up were used for antigen and antifilarial antibody assays. All blood specimens were collected by finger prick; venipuncture was not acceptable to the mothers of the children. Filarial antigen status was determined by the commercial Og4C3 ELISA kit (TropBio, Townsville, Australia). Serum samples were diluted 1∶10 in sample buffer and then assayed in duplicate according to the manufacturer's instructions as previously described [1]. Samples with antigen levels ≥128 units were considered to be positive. Antibody responses to Wb123, a *Wuchereria bancrofti* L3-specific antigen selected based upon its lack of cross reactivity with other filarial species, were measured using the highly sensitive LIPS (Luciferase Immunoprecipitation System) assay [16], [17]. A detailed description of the antigen is provided in the companion paper [17].

Serum samples were run in duplicate against a standard curve in order to control for plate to plate variability. Positive values were interpreted from the data based on a cutoff value, 10968 LU/ml, which was based on the responses of sera from 50 nonendemic persons using receiver operating characteristic (ROC) analysis.

Bm33, also known as Bm-AP-1, was included in the study based on previous reports that it was frequently recognized by sera from persons in LF-endemic areas [18]–[20]. The cloning and purification of recombinant Bm33 protein containing both an amino-terminal GST fusion and a carboxy-terminal 6× His tag have been previously reported [20]. Similarly, the cloning and purification of a recombinant major surface protein (WSP) from *Wolbachia* have been previously reported [21]. WSP was expressed as a 6× His-tagged dihydrofolate reductase (DHFR) fusion protein and was cleaved from the fusion partner using thrombin [21]. Bm14, also known as SXP-1 has been used extensively both as a diagnostic antigen and for monitoring LF programs [9]–[11], [22], [23]. For this work, the *Brugia malayi* Bm14 antigen coding sequence [22] was PCR amplified from an adult female cDNA library in Lambda Uni-Zap XR (National Institutes of Health/National Institute of Allergy and Infectious Diseases Filariasis Research Reagent Repository Center, Molecular Resources Division, Smith College, Northampton, MA) and was cloned into the *Bam*HI and *Eco*RI restriction endonuclease sites of pGEX 4T-2 expression vector (GE Healthcare, Piscataway, NJ) using previously described techniques [19]. The deoxyoligonucleotides used for PCR amplification were: 5′-CGC GGA TCC CAA AGA GAA GCA CAA TTA CCT CAG-3′ and 5′-GCG GAA TTC TTA TTG TGA ATT AAA TCC TTC CAA GAT-3′. Recombinant Bm14/GST protein was purified on a GST affinity column as directed by the manufacturer (GE Healthcare). Purity of the Bm14/GST recombinant protein was estimated to be >99% by polyacrylamide gel electrophoresis with Coomassie Blue staining.

Purified Bm14, Bm33, and WSP recombinant proteins (120 µg of protein for 12.5×10^6^ beads) were coupled at pH 7.2 to SeroMap beads (Luminex Corp., Austin, TX) as previously described [20]. Antibody responses to Bm14 and Bm33, and to WSP from Wolbachia were analyzed using a multiplex bead assay that incorporated 28 antigens, including malaria and vaccine antigens as well as antigens from a number of waterborne pathogens [20], [24]. Inclusion of nonfilarial antigens provided additional controls for sample integrity; i.e, responses to certain antigens (e.g., enterotoxigenic *E. coli* heat labile toxin Beta subunit and SAG2 of *Toxoplasma*) when positive, were consistent across samples from a given child. Thus, the absence of expected responses was considered evidence that samples were degraded or had been misnumbered. Data from these samples (13 from a total of 785 serum samples) were deleted. Responses to nonfilarial antigens will be reported elsewhere. All serum samples for the multiplex assay were diluted in PBS containing 0.05% BSA, 0.05% Tween 20, 0.02% sodium azide, 0.5% polyvinyl alcohol (PVA), 0.8% polyvinylpyrrolidone (PVP) to reduce the background reactivity [25]. Crude *E.coli* extract was added to the dilution at a final concentration of 3 µg/ml to decrease potential nonspecific binding of antibodies to residual *E.coli* proteins in purified recombinant proteins [20]. Samples were run in duplicate at a final serum dilution of 1∶400; antigen-coated beads were incubated with the samples for 90 minutes. Data are reported as the average of the median fluorescence intensity from duplicate wells minus the background from a serum blank run in parallel on each plate (MFI-bg). Thresholds for positive responses were defined based on the mean plus three standard deviations of the antifilarial antibody response of serum samples from nonendemic persons.

### Statistical analysis

Using the Kaplan-Meier (KM) method, failure rate probabilities were computed for age at time of initial response for each antibody. Cox proportional hazards model was used to investigate factors that influenced the age at which the initial antibody response occurred. Gender, infection status (both mother's and child's), neighborhood in which child resides, and study year were considered. Children with no response by the time of their last follow-up visit were considered censored in both the KM survival analysis and proportional hazards model.

Poisson regression was used to estimate seroconversion rates and their related confidence intervals. The nonparametric Kruskal Wallis test was utilized to test for differences in age at time of first sample and number of years of follow-up between the three neighborhoods.

## Results

Longitudinal studies were set up in Leogane neighborhoods to monitor antifilarial immune responses associated with exposure to LF and development of patent filarial infection. The median period of follow-up for the children in this study was 4.7 years, with the first sample collected at a median age of 1.4 years. Ninety-six (67.6%) of the one hundred and forty-two children were from the Bino neighborhood of Leogane, and boys (57.7%) outnumbered girls (42.3%) in the study population (Table 1). Median age at time of first sample was 0.9 years in Cada, 1.4 years in Bino, and 2.2 years in “other” locales. The difference was significant at p = 0.006. There was no difference, however, in the median number of years of follow up between children from different areas (p = 0.83).

**Table 1:** Characteristics of the Study Population.

Area	N (%)	Male	Median Age (Years) at Time of First Sample	Median Years of Follow-up
Bino	96 (67.6)	58 (60.4)	1.4	4.7
Cada	24 (16.9)	9 (37.5)	0.9	4.6
Other	22 (15.5)	15 (68.2)	2.2	4.7
Total	142 (100)	82 (57.8)	1.4	4.7

**Figure 1:**
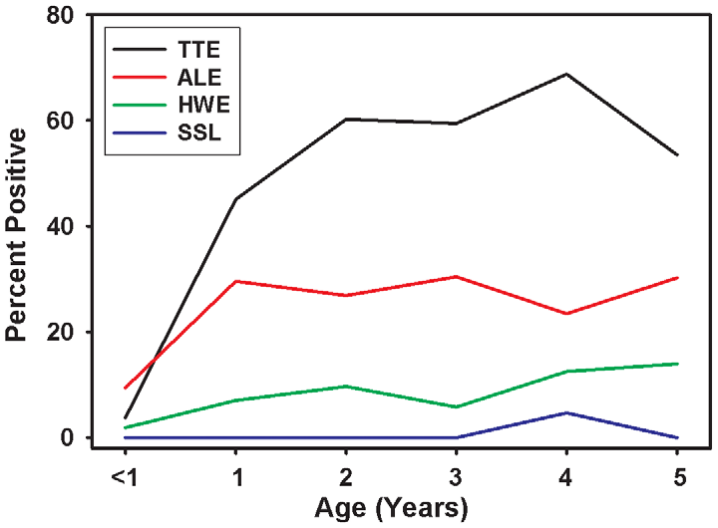
Prevalence of intestinal helminths. The prevalence of *Trichuris trichiura* eggs (TTE), *Ascaris lumbricoides* eggs (ALE), Hookworm eggs (HWE) and *Strongyloides stercoralis* larvae (SSL) was assessed in stools from children enrolled in the longitudinal cohort during the first 5 years of life.

Children were monitored periodically by nocturnal blood exam for microfilaremia and by microscopic examination of stool samples for intestinal parasites. Early infection and re-infection with intestinal helminths was a common occurrence throughout the study. The prevalence of *Trichuris*, *Ascaris*, hookworm and *Strongyloides* infection in children under the age of 5 is shown in Figure 1. By the age of 3, more than 59% and 30% of children were infected with *Trichuris* and *Ascaris*, respectively. Over the period of follow-up, hookworm prevalence increased dramatically in the community [26].

Nocturnal blood smears were prepared for microfilaremia assessment during each sampling period. The cumulative prevalence of microfilaremia in the children was 23.2% by the end of the study (Table 2); this prevalence should be considered a minimum estimate because of the small volume of blood (20 µl) examined. Serum samples were assessed for circulating filarial antigen using the Og4C3 ELISA to determine when children first became antigen positive; cumulative antigen prevalence was 56.3%. All microfilaria-positive children were also antigen-positive. The mean age at which children acquired *W. bancrofti* infection as assessed by microfilaremia and antigenemia was 6.3 and 4.3 years, respectively (Table 2).

**Table 2:** Summary of results (n = 142).

Test	Positive (%)	Mean Age (years) at time of first positive
Mf	30 (21.1)	6.4
Ag	80 (56.3)	4.3
Wb123	130 (91.6)	3.7
Bm14	135 (95.1)	3.4
Bm33	142 (100)	2.8
WSP	41 (28.9)	4.3

**Figure 2:**
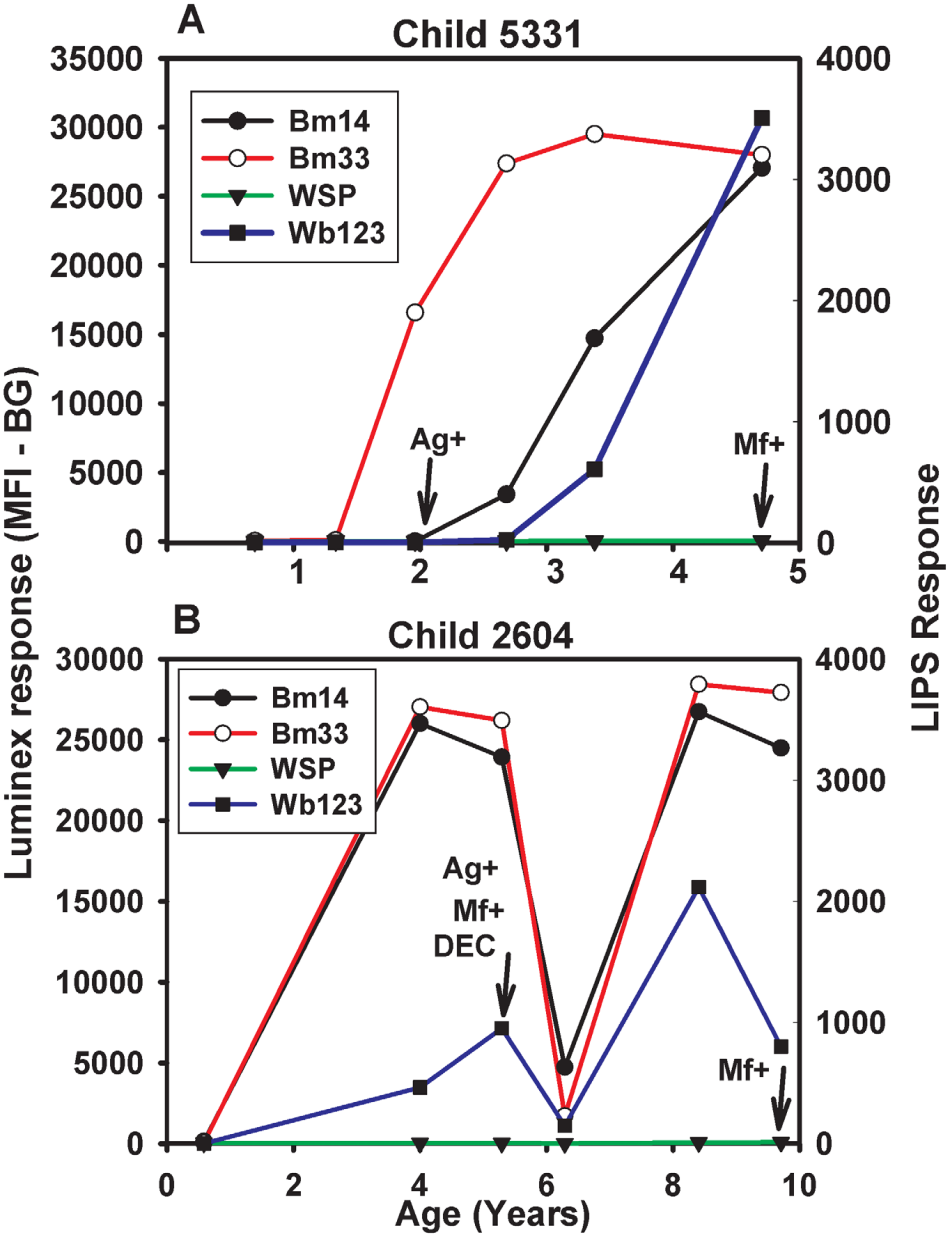
Representative antibody profiles. Antibody responses to Wb123, Bm14, BM33 and WSP were monitored in serum samples for child #5331 (panel A) and child #2604 (panel B). collected over time. First detection of antigenemia (by Og4C3 ELISA) and microfilaremia are indicated. Microfilaremic children were treated with DEC.

Antibody responses to Bm14, Bm33, and WSP antigens were measured using a multiplex assay platform. A novel antigen, Wb123, was measured using LIPS technology. Positive antibody responses for both techniques were defined based on cutoff values determined from nonendemic control samples. Representative plots from two children are shown in Fig. 2. Increases in antifilarial antibody to Wb123, Bm33 and Bm14 were noted in conjunction with (e.g., panel A) or prior to the detection of filarial antigen (panel B). Antibody to WSP was not detected in most children. Children were treated with DEC when microfilariae were detected and as previously reported [20], treatment often led to declines in levels of antibody against all the filarial antigens as seen in Figure 2, panel B.

**Figure 3:**
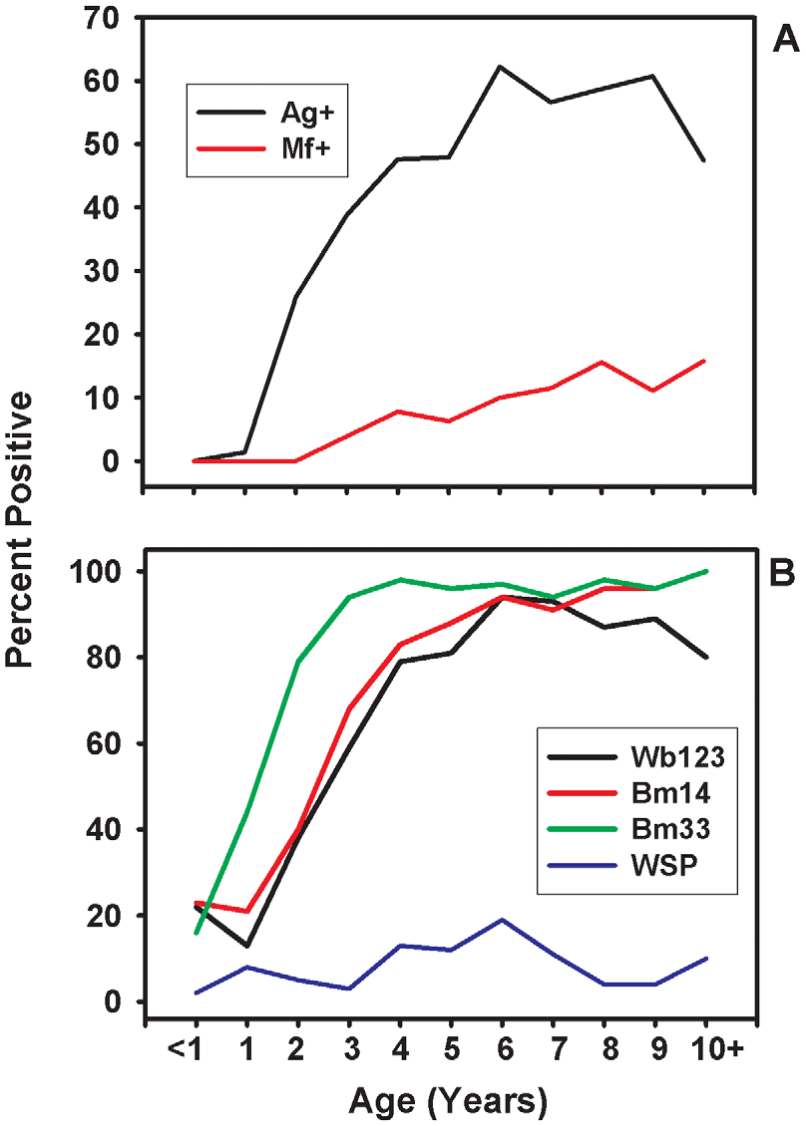
Age prevalence of microfilaremia, antigenemia and antifilarial antibody responses. Circulating filarial antigen and microfilaria prevalence are shown in the top panel A. Antibody responses to Bm14, Bm33, Wb123, and WSP filarial antigens are shown in the bottom panel B.

Age prevalence curves showing the profiles of circulating filarial antigen, microfilaria, and the antibody responses to Bm14, Bm33, Wb123, and WSP filarial antigens with age are shown in Figure 3. Responses to Bm14, Bm33, and Wb123 increased markedly between one and three years of age. Bm33 was the first antibody response to be detected in children with a mean age of incidence occurring at 2.8 years, followed by Bm14 (3.4 years), Wb123 (3.7 years), and WSP (4.3 years) (Table 2).

The longitudinal nature of the study provided an opportunity to analyze serocoversion rates. Over the course of the study, 55.6, 47.8, and 61.9% of children seroconverted to Bm14, Bm33, and Wb123, respectively. Only 23.9% of children developed responses to WSP and these responses were often transient in nature and unrelated to changes in antibody to the other filarial antigens. The rate of seroconversion was measured using total person years and was highest for Bm33, with 49.9 seroconversions per 100 person-years, followed by Bm14, Wb123, and WSP with seroconversion rates of 34.7, 31.0, and 5.5 per 100 person-years, respectively (Table 3).

**Table 3:** Seroconversion rates by antigen.

Antigen	Number of Seroconverters	Total # person days	Seroconversion Rate (per 100 years)	95% CI for rate	
Wb123	88	103705	31.0	24.9	37.9
Bm14	79	83212	34.7	27.6	43.0
Bm33	68	49793	49.9	38.9	62.7
WSP	34	224360	5.5	3.9	7.6

The correlation of children's infection status and antibody status at the end of the study is represented in Table 4. All of the children who were microfilaria-positive at the end of the study had antibody responses to Bm14 and Bm33 antigens, and 96.7% of these children were found to have a Wb123 response. Of the 80 children determined to be antigen-positive, 100, 100, and 97.5% had responses to Bm14, Bm33, and Wb123, respectively. Of children who were considered uninfected by antigen tests (Og4C3-negative) and microscopy (microfilaria-negative), 88.7, 100, and 83.9% had filarial antibody responses to Bm14, Bm33, and Wb123, respectively. The antibody prevalence was not significantly different by infection status for any of the filarial antigens.

**Table 4:** Antibody response by infection status.

Infection Status	Total	Bm14 N (%)	Bm33 N (%)	Wb123 N (%)
Ag+ and Mf+	30	30 (100)	30 (100)	29 (96.7)
Ag+	80	80 (100)	80 (100)	78 (97.5)
Ag−	62	55 (88.7)	62 (100)	52 (83.9)

**Figure 4:**
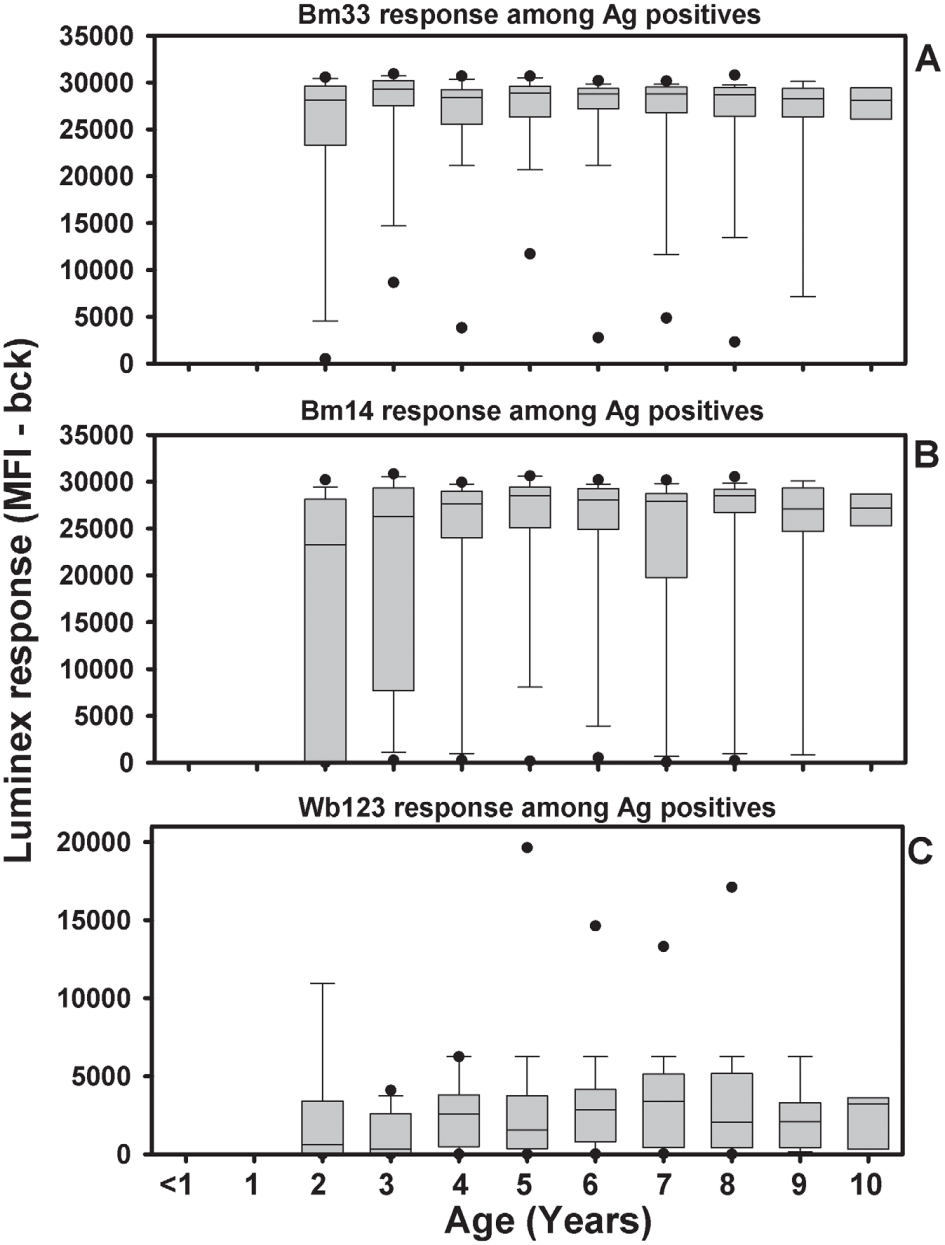
Quantitative changes in antibody among antigen-positive children. Shown are Bm33 (panel A), Bm14 (panel B), and Wb123 (panel C) antibody levels by age for antigen-positive children. In this plot, boxes represent the 25^th^–5^th^ percentile with the line in the box, the median. Whiskers represent the10^th^ and 90^th^ percentile and filled circles are 5^th^ and 95^th^ percentile. Note that Luminex unit values are specific to each antigen and should not be assumed to be equivalent across antigens.

**Figure 5:**
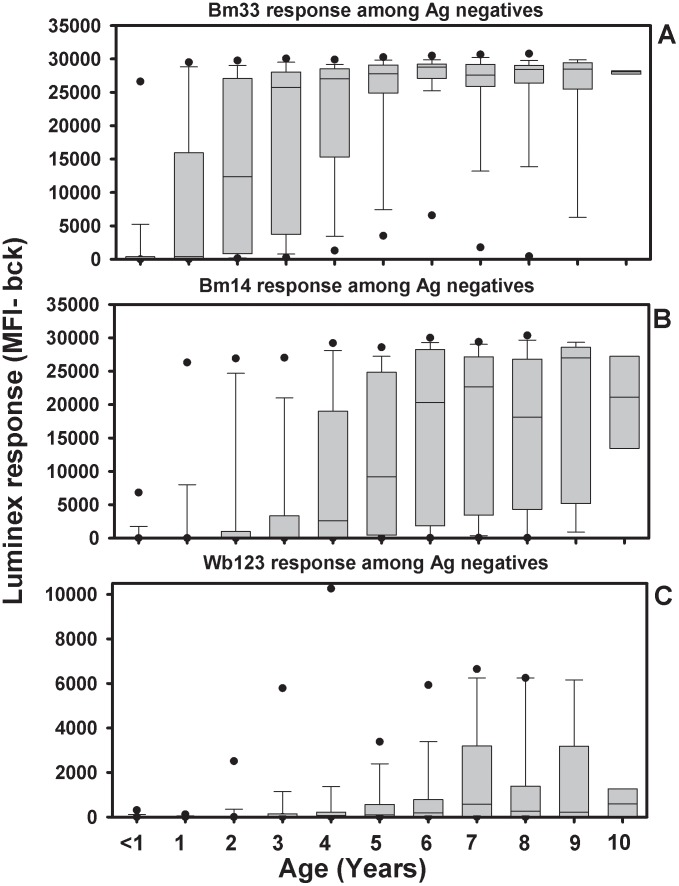
Quantitative changes in antibody among antigen-negative children. Shown are Bm33 (panel A), Bm14 (panel B), and Wb123 (panel C) antibody levels by age for antigen-negative children. In this plot, boxes represent the 25^th^–5^th^ percentile with the line in the box, the median. Whiskers represent the10^th^ and 90^th^ percentile and filled circles are 5^th^ and 95^th^ percentile.

Quantitative analyses of the antibody responses of antigen-positive and antigen-negative children are shown in in Figures 4 and 5, respectively. Antigen-positive children had levels of anti-Bm33 and anti-Bm14 antibody that were at or near the maximum level of the assay at the serum dilution tested across all ages; however, Wb123 responses were lower than the peak responses. Among antigen-negative children, anti-Bm33 responses increased with age, reaching maximal values by age 5. Similar increases in antibody levels with age were noted for Bm14 and Wb123, but antibody levels did not reach assay maximums for either antigen. A Cox proportional hazards model was generated to analyze factors influencing antibody responsiveness, including gender, child's infection status, maternal infection status, community of residence, and study year. The analysis for Bm14 is shown in Table 5. Bm14 responses were influenced by gender with females responding at an earlier age than males, but not by antigen status, maternal infection status, or community of residence. Children who were sampled during the early study period of 1990–1995 were significantly more likely to develop a Bm14 antibody response than children sampled during the later study period (1996–1999) (p = 0.0036). A similar result was seen for Wb123 and WSP, but not Bm33 (p = 0.37).

**Table 5:** Cox proportional hazards model for initial Bm14 antibody response.

Parameter		DF	Parameter estimate	Standard error	Chi-square	Pr>Chisq	Hazard Ratio	Confidence Limits	
Sex	Female	1	0.37743	0.18822	4.0211	0.0449*	1.459	1.009	2.109
Ag Status	Positive	1	−0.09595	0.19105	0.2523	0.6155	0.909	0.625	1.321
Ag Status Mom	Positive	1	0.09241	0.18636	0.2459	0.6200	1.097	0.761	1.58
Area	Bino	1	0.00518	0.25890	0.0004	0.9840	1.005	0.605	1.67
Area	Cada	1	0.26453	0.32127	0.6780	0.4103	1.303	0.694	2.445
Study year	1990–1995	1	0.64043	0.22017	8.4609	0.0036*	1.897	1.232	2.921

## Discussion

Understanding the development of antifilarial immunity in children exposed to filarial infection can help inform the strategies for monitoring LF elimination programs. Our results confirm that antifilarial antibodies are early markers of infection and develop before circulating filarial antigen, the current marker used by programs both for making decisions about stopping MDA and post-MDA surveillance [27]. In a setting of intense transmission, more than one-half of the children developed infection with *W. bancrofti* over the period of follow-up and nearly all developed antibody responses to defined recombinant antigens. We have previously shown that children in these Haitian communities develop antifilarial antibodies as early as 2 year of age and that antifilarial IgG1 responses preceded IgG4 responses; however these assays were carried out with crude parasite extracts and the utility of these assays was limited by the lack of specificity of the antibody responses [1].

Although intense transmission environments are not the most stringent settings for testing the sensitivity of the antibody tests because of elevated antigen loads, it is noteworthy that virtually all microfilaria-positive children developed antibody responses to each of the three filarial antigens that we tested (Table 2). Indeed, antibody responses were nearly universal among children in these neighborhoods. To varying extents, the different antibody responses typically developed before antigenemia, providing additional support for the concept that antibody responses will provide useful tools for monitoring LF programs. Bm33 was the first antigen to elicit an antibody response and was detected on average more than 1 year before the other antibody responses. Levels of Bm33 antibody were elevated among both antigen-negative and antigen-positive children by four years of age, indicating that antigenemia is not a requirement for robust antibody responses (Fig. 3). Bm33 is homologous to immunodominant antigens from other filarial parasites, suggesting that it may have broad utility as a monitoring tool [28], [29]; however, its specificity has not been defined rigorously, and it is possible that cross-reactivity could occur in persons infected with other filarial worms such as *Loa loa* or *Onchocerca volvulus*. Bm14 responses generally developed after Bm33, but on average about one-half year before the development of antigenemia. Bm14 is also known to be recognized by sera from patients with other filarial infections [9]. In contrast, Wb123 is exquisitely specific, an important advantage in geographic areas where multiple filarial species are transmitted ([17]. Among children enrolled in our study, responses to Wb123, an antigen expressed in infective stage larvae, developed after Bm33, originally cloned from an adult worm cDNA library. These results argue that quantitative aspects of exposure to specific antigens may be important in defining the timing and magnitude of the antibody response. Additional operational validation of these tests is needed to define their utility in the programmatic context, especially in the later stages of the program when infection prevalence has declined.

Antibody responses to WSP were uninformative. Only a small proportion of children mounted detectable responses to WSP, responses were generally low and were not obviously correlated with responses to the other filarial antigens. We have previously hypothesized that WSP responses were related to adult worm death [21], but our current results do not shed any light on this hypothesis.

The significance of an antifilarial antibody response in children has not been fully defined. Historically, such responses have been considered markers of exposure rather than infection [1], [9], [10]. Recent evidence suggests that Bm14 and Bm33 antibody responses are, in fact, infection markers, since antibody responses declined significantly in antigen-negative antibody-positive children treated with DEC, but not with placebo [20]. These observations have important implications for the interpretation of antibody responses among children at terminal phases of the LF elimination program. Additional research is needed to determine whether antibody responses are specific markers of infection; if so, assessing the sero-reversion of antibody responses may be useful as a monitoring strategy.

In terms of monitoring tools, it is possible that antibody responses could be used as a measure of transmission intensity as has been described in malaria [30]. If so, the shape of age prevalence curves should be useful as a measure of transmission intensity. Similarly, the relationship between quantitative measures of the antifilarial antibody response and transmission intensity should be further explored. In our study, we were not able to address these issues because of the limited heterogeneity in infection and antibody reactivity across communities.

Our study was originally set up to address risks factors for acquisition of *W. bancrofti* in children, based in part on earlier epidemiologic evidence that infection clustered in families and that the infection status of children was influenced by maternal infection status [31]. In additional analyses, we did not see any relationship between maternal infection status and the age at which antibody responses developed in this cohort of children (Table 5). Similarly, although we hypothesized that the early acquisition of intestinal helminth infections would influence the acquisition of infection and development of antifilarial antibody responses, we did not see such a relationship (data not shown). The absence of a detectable relationship between intestinal helminth infection and antifilarial antibody responses may be a reflection of the relatively low infection intensities typically found in Leogane for the intestinal worms [32]. Additional studies in other sites are needed to further investigate this relationship. It is also important to note that our selection of children for the serological sub-study was not random; only children with 5 or more follow-up visits were included. We are not able to say whether or not this bias influenced the analyses.

Our study has some limitations that may significantly impact our conclusions. We were not always able to follow up the children as frequently as we had planned, particularly during the embargo period when travel to Haiti was more difficult. For ethical reasons, we also treated all microfilaria-positive persons who were identified during our community surveys. Although the number of persons treated during any single time period was relatively small (as a proportion of the total population), the cumulative number of people treated did increase over time. Our antibody analyses suggested that the period of sample collection did influence the antibody response in children and we believe that the treatment we offered to microfilaremic persons is the most likely explanation for this (Table 5).

Finally, although our results argue for an expanded role for antibody testing as part of the monitoring strategy for the LF program, it is clear that a great deal of additional work is needed to validate the use of these tests in low prevalence settings. In principle, this could be done easily in the context of planned transmission assessment surveys as countries try to determine when to stop MDA. Depending on the antigen used, it is likely that the numbers of antibody positive children will exceed the number of antigen positive children. Similarly, the choice of test format will also influence antibody prevalence; both LIPS and multiplex tests may be more sensitive than conventional ELISA or rapid test formats. In either case, this may create an additional monitoring option during the post-MDA surveillance period as proposed previously [10], [33], [34]. New WHO surveillance recommendations are based on carrying out repeated transmission assessment surveys (TAS) [27]; however, repeat TAS surveys are not powered to detect a significant change in the number of antigen positive children. Perhaps the detection of larger numbers of antibody-positive children as part of the TAS will allow surveys to be powered to detect significant changes in antibody prevalence. Similarly, quantitative assessments of antibody levels may also provide additional insight into changes in transmission intensity. Comprehensive analyses of antibody responses following MDA are urgently needed.

### Footnotes

Use of trade names is for identification only and does not imply endorsement by the Public Health Service or by the U.S. Department of Health and Human Services. The findings and conclusions in this report are those of the authors and do not necessarily represent the official position of the Centers for Disease Control and Prevention.

## Supporting Information

Checklist S1 **Strobe checklist.**
(DOC)
